# Characterization of the *hupSL *promoter activity in *Nostoc punctiforme *ATCC 29133

**DOI:** 10.1186/1471-2180-9-54

**Published:** 2009-03-11

**Authors:** Marie Holmqvist, Karin Stensjö, Paulo Oliveira, Pia Lindberg, Peter Lindblad

**Affiliations:** 1 Department of Photochemistry and Molecular Science, The Ångström Laboratories, Uppsala University, Box 523, SE-751 20 Uppsala, Sweden; 2 Department of Plant and Microbial Biology, University of California, Berkeley, CA 94720-3102, USA

## Abstract

**Background:**

In cyanobacteria three enzymes are directly involved in the hydrogen metabolism; a nitrogenase that produces molecular hydrogen, H_2_, as a by-product of nitrogen fixation, an uptake hydrogenase that recaptures H_2 _and oxidize it, and a bidirectional hydrogenase that can both oxidize and produce H_2_.*Nostoc punctiforme *ATCC 29133 is a filamentous dinitrogen fixing cyanobacterium containing a nitrogenase and an uptake hydrogenase but no bidirectional hydrogenase. Generally, little is known about the transcriptional regulation of the cyanobacterial uptake hydrogenases. In this study gel shift assays showed that NtcA has a specific affinity to a region of the *hupSL *promoter containing a predicted NtcA binding site. The predicted NtcA binding site is centred at 258.5 bp upstream the transcription start point (tsp). To further investigate the *hupSL *promoter, truncated versions of the *hupSL *promoter were fused to either *gfp *or *luxAB*, encoding the reporter proteins Green Fluorescent Protein and Luciferase, respectively.

**Results:**

Interestingly, all *hupsSL *promoter deletion constructs showed heterocyst specific expression. Unexpectedly the shortest promoter fragment, a fragment covering 57 bp upstream and 258 bp downstream the tsp, exhibited the highest promoter activity. Deletion of the NtcA binding site neither affected the expression to any larger extent nor the heterocyst specificity.

**Conclusion:**

Obtained data suggest that the *hupSL *promoter in *N. punctiforme *is not strictly dependent on the upstream NtcA cis element and that the shortest promoter fragment (-57 to tsp) is enough for a high and heterocyst specific expression of *hupSL*. This is highly interesting because it indicates that the information that determines heterocyst specific gene expression might be confined to this short sequence or in the downstream untranslated leader sequence.

## Background

In cyanobacteria there are three enzymes directly involved in hydrogen metabolism; nitrogenase, uptake hydrogenase and bidirectional hydrogenase [1-3]. During nitrogen fixation, nitrogenase evolves molecular hydrogen (H_2_) as a by-product. The uptake hydrogenase consumes the H_2 _to recapture energy, thereby preventing losses from the cells, while the bidirectional hydrogenase has the capacity to both evolve and consume H_2 _[1-3]. The exact function of the bidirectional hydrogenase is unknown, but it has been proposed both to play a role in fermentation and to act as an electron valve during photosynthesis [2]. The uptake hydrogenase has been found in all filamentous nitrogen fixing (N_2 _fixing) cyanobacterial strains examined so far. The enzyme is expressed in the heterocysts (cells specialized for nitrogen fixation) under conditions of combined nitrogen starvation and is functionally connected to nitrogen fixation [1]. Cyanobacterial uptake hydrogenase consists of at least a small subunit, HupS, and a large subunit, HupL and the genes encoding the small and the large subunit, *hupS *and *hupL*, have been identified in a number of cyanobacteria [2,4-6]. Relatively little is known about the regulation and maturation of the uptake hydrogenases in cyanobacteria and the knowledge is mainly based on studies made in *Escherichia coli*. The active sites in the large subunits of hydrogenases are very complex and require a set of accessory proteins for their correct assembly and folding, which in *E. coli *are encoded by *hypA*-*F *[7,8]. Homologues of these genes are present in cyanobacteria [2,9]. In addition, recently a set of genes within the extended *hyp*-operon was suggested to be involved in the maturation of the small subunit of the cyanobacterial uptake hydrogenase [10].

*Nostoc punctiforme *ATCC 29133 (*N. punctiforme*) is a filamentous dinitrogen fixing cyanobacterium that was originally isolated from the coralloid roots of the cycad *Macrozamia *[11]. This strain contains a nitrogenase and an uptake hydrogenase, but lacks the bidirectional hydrogenase [12]. In 1998 *hupS *and *hupL *were identified and characterized in *N. punctiforme *[13]. Later on, transcriptional analyses showed that *hupS *and *hupL *are transcribed as one operon thereby sharing the same promoter [14]. Furthermore, a transcription start point (tsp) was identified 259 bp upstream the translation start of *hupS*, with a putative transcription terminator downstream of *hupL *and a hairpin formation in the intergenic region between *hupS *and *hupL *[14]. Upstream of this transcription start point some putative regulatory promoter elements were identified, among them a possible binding site for the transcription factor NtcA [14]. NtcA belongs to the CAP family of transcriptional regulators, and is a global nitrogen regulator in cyanobacteria [15,16]. In *N*. *punctiforme *as well as in *Nostoc *sp. strain PCC 7120, NtcA is necessary for heterocyst differentiation [17,18]. NtcA has also been identified as a regulator of several other genes whose expression is either induced or repressed during heterocyst differentiation or in the mature heterocysts [15,16]. In other bacteria such as *Rhodobacter capsulatus*, *Ralstonia eutropha*, *Bradyrhizobium japonicum*, and *Rhodopseudomonas palustris hupSL *transcription is upregulated in the presence of H_2 _by the two component signal transduction system HupT/HoxJ and HupR/HoxA [19-23]. This regulatory system is functionally connected to the activity of the H_2 _sensing hydrogenase HupUV/HoxBC [19-23]. In the photosynthetic bacterium *Thiocapsa roseopersicina *however, the regulation of the uptake hydrogenase by HupR seems to be independent of the presence or absence of H_2_, and the genes encoding HupT and HupUV are not expressed [24]. The regulation of *hupSL *by the redox sensing two component signal transduction system consisting of RegA and RegB has been discovered in *R. capsulatus *[25]. Furthermore, regulation by the nitrogen fixation regulatory protein, NifA, has been reported for *Rhizobium leguminosarum *bv. Viciae [26,27]. The function of NifA in activating transcription of *hupSL *in *R.legminosarum *is stimulated by the integration host factor (IHF) which facilitates contacts between NifA and the polymerase by binding to and bending the *hupSL *promoter [27,28].

The uptake hydrogenase in filamentous dinitrogen fixing cyanobacteria is expressed in the heterocysts [29,30]. The expression has been shown to be regulated at the transcriptional level in *Nostoc muscorum *[31], *Anabaena variabilis *ATCC 29413 [32], *N. punctiforme *[9] and *Nostoc *sp. strain PCC 7120 [33]. A transcript is detectable about 24 h after transition from non-N_2 _fixing to N_2 _fixing conditions in *A*. *variabilis *[32] and *N*. *muscorum *[31]. Even though no sensor hydrogenase has been found in cyanobacteria, an upregulated transcription level was detected in the presence of H_2 _in *N. punctiforme *[33,34] and *N.muscorum *[34]. Interestingly, this upregulation of *hupSL *expression in response to H_2 _was not observed in *A.variabilis *[35]. Putative binding sites for NtcA have, in addition to N. punctiforme [36], also been identified in the *hupSL *promoter of *Nostoc *sp. PCC 7422 [37], *Lyngbya majuscula *CCP 1446/4 [38], *Gloeothece *sp. ATCC 27152 [39] and *A.variabilis *[35] and NtcA was also shown to bind to the predicted binding sites [35,38,39]. Furthermore, putative IHF binding sites have been identified in the promoter region of *N. punctiforme *[14] and *L. majuscula *CCAP 1446/4 [38]. Based on what is known about the regulation of *hupSL *transcription in cyanobacteria and other bacteria, a regulation of the *hupSL *operon in *N. punctiforme *by NtcA is not unlikely.

In this study the binding of purified NtcA to the putative recognition site, previously identified in the *hupSL *promoter, was examined. The result showed that NtcA does bind to the *hupSL *promoter in *N. punctiforme*, even though the *hupSL *transcription seems to be not strictly dependent on the NtcAcis element identified. Furthermore, regulatory regions in the *hupSL *promoter in *N. punctiforme *were mapped by fusing truncated sequences of the *hupSL *promoter to the either *gfp *or *luxAB*, encoding the reporter proteins GFP (Green Fluorescent Protein) and Luciferase respectively. All the longer promoter constructs showed heterocyst specific expression and unexpectedly the shortest promoter construct, a 316 bp DNA fragment stretching from 57 bp upstream the tsp to the translation start point, conferred not only the highest transcription levels but also retained the heterocyst specificity of the expression. This finding is of great interest since it indicates that the information required for a gene to be expressed in heterocysts only might be located in this short DNA sequence.

## Methods

### Strains and culture conditions

*Nostoc punctiforme *ATCC 29133 cultures were grown in BG11_0 _medium [40] either in 100 ml Erlenmeyer flasks on a shaking table or on plates containing BG11_0 _medium solidified by 1% noble agar (Difco). Larger volumes of *N. punctiforme *cultures were grown in 1 L Erlenmeyer flask containing BG11_0 _medium under continuous stirring and sparging with air. All cultures were grown at 25°C at a continuous irradiance of 40 μmol of photons m^-2 ^s^-1 ^(29). For cultures treated by sonication or were electroporated, the BG11_0 _medium was supplemented with 5 mM MOPS (pH 7.8) and 5 mM NH_4_Cl as a combined nitrogen source. 10 μg/ml ampicillin was used for selection of positive clones after electroporation with the vector constructs. All cloning was done using *Escherichia coli *strain DH5α grown at 37°C in Luria broth (LB) liquid medium [41], supplemented with 100 μg/ml ampicillin, and on plates containing LB medium solidified with 1% agar and supplemented with 100 μg/ml ampicillin.

### PCR, DNA sequencing and sequence analysis

Genomic DNA was isolated from *N. punctiforme *cultures as previously described [12]. The concentration was determined by absorbance measurements using Cary Win UV (Varian). PCR amplifications were carried out using the high fidelity DNA polymerase Phusion (Finnzymes), according to manufacturer's protocol, in a GeneAmp PCR system 2400 (Applied Biosystem). The primers used in this work are listed in Table 1. All primers were designed using the Primer3 program http://frodo.wi.mit.edu/cgi-bin/primer3/primer3_www.cgi and blasted against the *N. punctiforme *genome [42] (JGI Microbial genomes, http://genome.jgi-psf.org/mic_home.html), or in the case of sequencing primers against their corresponding vector sequence (Table 1), to check their specificity. Secondary structure of the primers was analysed with the Primer design utility program http://www.cybergene.se/primerdesign/. Amplified DNA fragments were isolated from agarose gels using the GFX PCR DNA and Gel Band Purification Kit (GE Healthcare), following the manufacturer's instructions. Sequencing reactions were performed by Macrogen Inc. and computer-assisted sequence analyses were performed using BioEdit Sequence Alignment Editor Version 7.0.5.3.

**Table 1:** Plasmids and primers used in the present study

Plasmids	Reporter gene	Reference
pSUN202	*gfp*	(2)
pA-gfp	*gfp*	This study
pB-gfp	*gfp*	This study
pC-gfp	*gfp*	This study
pD-gfp	*gfp*	This study
pE-gfp	*gfp*	This study
pP*prbcL-gfp*	*gfp*	This study
pLR1	*luxAB*	P Lindberg, unpublished
pA-*lux*	*luxAB*	This study
pB-*lux*	*luxAB*	This study
pC-*lux*	*luxAB*	This study
pD-*lux*	*luxAB*	This study
pE-*lux*	*luxAB*	This study
pP*prbc-Llux*	*luxAB*	This study

**Primers**	**Sequence 5'-3'**	**Restriction site**

**GFP**		
CLONING		
A GFP forward	CGCGGTACCAGGCTTCGAGTCCTTTAGGC	*Kp*nI
B GFP forward	CGCGGTACCTCAATCCCCTAAATTG	*Kpn*I
C GFP forward	CGCGGTACCTTCTAAAATTCTAGGGGGAAATTG	*Kpn*I
D GFP forward	CGCGGTACCGACCTGACACAACGCAGTTC	*Kpn*I
E GFP forward	CGCGGTACCTCACCTTTAAAATCTTAGCCCATT	*Kpn*I
P*hupS *GFP reverse 1	CGCGAATTCGGGCTAGGTGTTTTTGTATTGT	*Eco*RI
P*hupS *GFP reverse 2	CGCCAATTGGGGCTAGGTGTTTTTGTATTGT	*Mun*I
P*prbcL *GFP forward	CGCGGTACCATCGGGCAAGGATTCT	*Kpn*I
P*prbcL *GFP reverse	CGCGCAATTGATTTTATCCTTCCCTGAAAT	*Mun*I
CONFIRMATION		
pSUN202 seq primer forward	CAAGTAGCGAAGCGAGCAG	-
pSUN202 seq primer reverse	TGGGACAACTCCAGTGAAAA	-
		
**LUCIFERASE**		
CLONING		
A lux forward	CATCTCGTAGGCCCGGTAAT	-
B lux forward	GCGCGAATTCTCAATCCCCTAAATTG	*Eco*RI
C lux forward	GCGCGAATTCTAAAATTCTAGGGGGAAATTG	*Eco*RI
D lux forward	GCGCGAATTCGACCTGACACAACGCAGTTC	*Eco*RI
E lux forward	GCGCGAATTCACCTTTAAAATCTTAGCCCATT	*Eco*RI
P*hupS *lux reverse	GCGCGGTACCGGGCTAGGTGTTTTTGTATTGT	*Kpn*I
Pp*rbc *lux forward	GCGCCAATTGCATCGGGCAAGGATTCT	*Mun*I
Pp*rbc *lux reverse	GCGCGGTACCGGCTTGATACCCAGACTTGC	*Kpn*I
CONFIRMATION		
pLR1 seq forward	AATACCGCACAGATGCGTAA	-
pLR1 seq reverse	CCAATTAGCGAATAGAGCTT	-
		
**EMSA**		
GS forward	GCGCGAATTCCCAATACCTAATACTTAATCCTC	-
GS reverse	GCGCTCTAGATAACAAAGTTAGGTCTTAA	-

Plasmids and primer oligonucleotides designed and used to amplify DNA fragments by PCR for vector construction, confirmation, and generation of cloned promoter fragments. Oligonucleotides designed and used for EMSAs are also shown. Restriction sites inserted in the cloning primers are listed.

### Electrophoretic Mobility Shift Assays (EMSA)

The *N*. *punctiforme hupSL *promoter region, -370 to -151 bp, relative to the transcriptional start point, was amplified with the primers GS Forward and GS Reverse (see Table 1). The 335 bp and the 229 bp DNA fragments used as unspecific, negative controls were amplified by PCR using pQE-30 (Qiagen) and pBluescript SK+ (Stragene) as template, respectively [43,44]. The *E.coli *BL21 (pCSAM70, pREP4) [45], kindly provided by Professor Enrique Flores (University of Seville, Spain) was used to overexpress the *Nostoc *sp. strain PCC 7120 NtcA fused to a histidine tag. NtcA from *Nostoc *PCC 7120 shares 100% amino acid identity with NtcA from *N. punctiforme *and was therefore used in the experiments. The His_6_-NtcA was purified by using Ni-nitrilotriacetic acid (Ni-NTA) Superflow resin column (Qiagen), according to the manufacturer's instructions. Binding assays were carried out as described [46] with 80 to 100 ng of DNA fragments and different amounts of purified NtcA (0–240 ng). After incubation at 30°C for 30 min, the reaction mixtures were separated by electrophoresis on a 6% (w/v) polyacrylamide non-denaturing gel and the relative positions of the DNA were visualized by staining the gel with ethidium bromide.

### Construction of the *hupSL *promoter deletions fused to *gfp*

To ensure correct orientation of the PCR generated promoter fragments when cloned in the self replicative, *gfp *containing, vector pSUN202 [47] (Table 1) restriction sites were included in the primers. A *Kpn*I site was added to the 5' end of the forward primers (A-E GFP forward, P*prbcL *GFP forward), while an *Eco*RI site or a *Mun*I site were added to the 5' end of the reverse primers (P*hupS *GFP reverse 1 and P*hupS *GFP reverse 2, P*prbcL *GFP reverse respectively) (Table 1). After PCR amplification, the products were digested with *Kpn*I/*Eco*RI (promoter fragments B-E) or *Kpn*I/*Mun*I (promoter fragments A and P*prbcL*) and subcloned upstream the *gfp *gene into a Shrimp Alkaline Phosphatase (SAP) treated, *Kpn*I/*Eco*RI digested, pSUN202 to give plasmid pA-*gfp *to pE-*gfp*, pP*prbcL-gfp*. The vector pSUN202 was kindly provided by Professor Michael Summers, California State University, Northridge, US. All enzymes used were from Fermentas and the ligations were made using Quick ligase (NEB). Correct cloning of all promoter fragments to pSUN202 were confirmed by sequencing using pSUN202 seq forward and pSUN202 seq reverse primer (Table 1). Both primers anneal to sites present within the original vector, pSUN202.

### Construction of the *hupSL *promoter deletions fused to *luxAB*

To ensure correct orientation of the PCR generated promoter fragments when cloned into the self replicable, *luxAB *containing vector pLR1 (Pia Lindberg, unpublished) (Table 1) restriction sites were included in the primers. An *Eco*RI or a *MunI *site was added to the 5' end of the forward primers (B-E lux forward and P*prbcL *lux forward respectively), and a *Kpn*I site to the 5' end of the reverse primer (P*hupS *lux reverse, P*prbcL *lux reverse) (Table 1). Primer A lux forward did not contain any restrictions site. Instead an intrinsic *Mun*I site in the resulting PCR product, (using A lux forward and P*hupS *lux reverse) was used for further cloning. After PCR amplification, the products were digested with *Eco*RI/*Kpn*I (promoter fragments B-E) or *Mun*I/*Kpn*I (promoter fragments A, P*prbcL *lux) and subcloned upstream *luxAB *into a SAP treated *Kpn*I/*EcoR*I digested pLR1 to give plasmids pA-*lux *to pE-*lux *and pP*prbcL-lux*. All enzymes used were from Fermentas and the ligations were made using Quick ligase (NEB). Correct cloning for all plasmids were confirmed by sequencing, using pLR1 seq forward and reverse primer (Table 1). Both primers anneal to sites present within the original vector, pLR1.

### Transformation of *N. punctiforme *cells and selection of positive clones

500 ml cell culture were harvested 3 days after inoculation and concentrated by centrifugation. The filaments were broken by sonication (Vibra cell VC 130, Sonics,) for 3 × 30 s (1 pulse/s, 20 kHz) to generate a culture with more single cells to allow for better segregation and selection of positive clones. The cell suspension was kept on ice for 30 s between the intervals. Chlorophyll *a *was extracted with 90% methanol and absorbance read against 665 nm using a Cary Win UV (Varian). The concentration of Chlorophyll *a *was determined using the extinction coefficient of 78.74 l g^-1^cm^-1 ^[48].

The vector constructs (pA-E, p1–5, pP*prbcL-gfp *and pP*prbcL-lux*) were transferred to *N. punctiforme *by electroporation. Overnight cultures of sonicated *N. punctiforme *cells were harvested by centrifugation and washed four times in sterile distilled water. The cells were then concentrated by centrifugation and diluted to a concentration of 50–100 μg Chl *a*/ml. 10 μg plasmid DNA dissolved in sterile distilled water were added to ice-cooled microcentrifuge tubes followed by 40 μl of concentrated cell culture. The cooled cell suspensions were transferred to an ice-cooled electroporation cuvette (2-mm electrode gap, Eppendorf) and exposed to a single electrical pulse. The pulse was delivered by a Gene-Pulser Xcell Microbial System (Bio-Rad Laboratories) set at 25 μF, 300 Ω and 1.6 kV. Immediately following the discharge, the suspensions were cooled on ice for about 5 min and thereafter transferred to culture flasks, containing ammonium supplemented growth medium, and left over night to recover. The cells were harvested and plated on ammonium supplemented, ampicillin containing plates. The plates were kept at low illumination (2–3 μmol of photons m^-2 ^s^-1^) and after 2 to 3 weeks of selection, positive colonies were picked and transferred to liquid medium supplemented with ammonium and ampicillin as detailed above. When the colonies had adjusted to the transition from growing on plates to liquid medium they were kept at standard illumination and transferred to plain growth medium to develop heterocysts. The constructs in the transformed cultures were confirmed by colony PCR. The primers used for the colony PCR (pSUN202 seq primer forward and reverse) anneal to the vector sequences flanking the inserted promoter region and hence the product spans the full length of the insert (Table 1).

### Fluorescence and luminescence measurements

**Figure 1:**
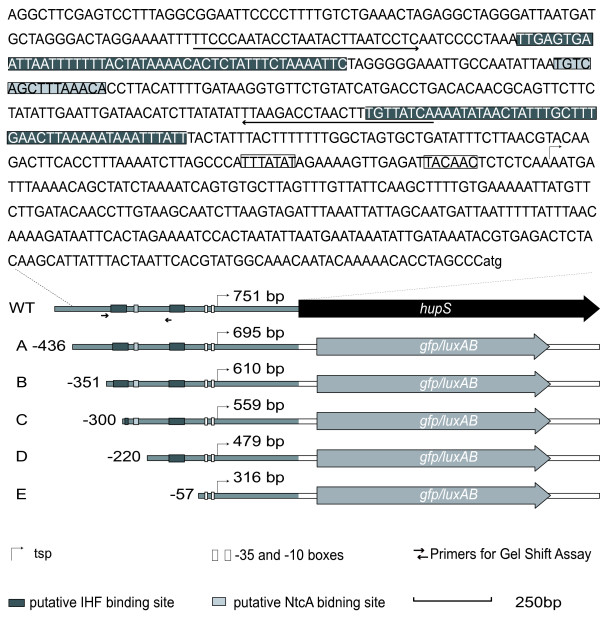
***hupSL *and its promoter region in *Nostoc punctiforme *ATCC 29133**. Detailed view of the nucleotides in the *hupSL *promoter region. Putative binding sites for regulatory proteins (IHF and NtcA), the transcription start point and the -35 and the – 10 boxes are marked [14]. Primers used for gel shift assay (see Fig. 2) are shown as arrows in the figure. Below the *hupSL *promoter sequence, the intergenic region between Npun_R0367 and *hupS *together with *hupS *are shown (WT). Furthermore, the five promoter deletion constructs, where truncated versions of the *hupSL *promoter have been coupled to *gfp *or *luxAB*, are also shown (A to E). Total length of the promoter fragments and starting position relative to transcription start point are indicated. The grey lines symbolise the *hupSL *promoter sequence, and the white lines symbolise the DNA sequence belonging to the vector used for the constructs, pSUN202.

Fluorescence emission of GFP was measured from whole cells (100 μl *N. punctiforme *culture at a concentration of 30 μg Chl *a*/ml) with an excitation wavelength of 488 nm and an emission wavelength of 520 nm using a Molecular Imager PharosFX Plus (Bio-Rad Laboratories). Luminescence from luciferase activity was induced by the addition of the substrate Decanal (n-Decyl Aldehyde, Sigma) to the cyanobacterial suspension. To 100 μl *N. punctiforme *culture (at a concentration of 30 μg Chl *a*/ml) 5 μl of a Decanal mixture was added. The mixture consisted of 7.8 μl Decanal, 500 μl Methanol (Fluka) and 500 μl distilled water. Light emission was monitored with a Molecular Imager ChemiDoc XRS System (Bio-Rad Laboratories). Fluorescence and luminescence measurements were performed at room temperature. Measurement data was corrected to the background (cells containing empty vector) and normalized to the chlorophyll *a *concentration of the samples. All measurements within one experiment were made in triplicate and performed at least three times using two independent clones. The clones containing the constructs pP*prbcL-gfp *and pP*prbcL-lux *were used as positive controls. Localization of GFP fluorescence was viewed in a fluorescence microscope (Leica DMRXE, Leica microsystems) with an excitation wavelength of 460–500 nm and an emission wavelength of 512–542 nm. The clone containing the construct pP*prbcL-gfp *was used as a positive control for localization of GFP fluorescence in vegetative cells. The micrographs were constructed by merging the DIC image with the corresponding fluorescence image for all promoter constructs (A to E, see Fig. 1) and the control construct p*PrbcL-gfp *in Photoshop CS2. The green color in the micrographs has been enhanced digitally to make the pictures clearer. The degrees of enhancement of green color were different for different constructs and hence no quantitative measurements could be done.

## Results

### Binding of NtcA to the *hupSL *promoter

**Figure 2:**
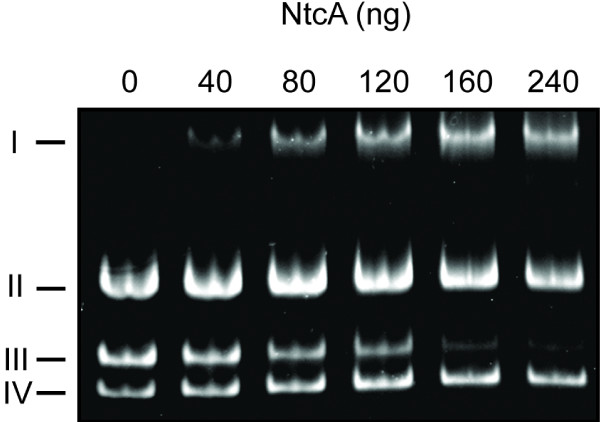
**Electrophoretic Mobility Shift Assays (EMSA)**. EMSA carried out with NtcA from *Nostoc *sp. strain PCC 7120 (overexpressed in *Escherichia coli *and purified before use) and the *Nostoc punctiforme *ATCC 29133 *hupSL *promoter region harbouring the putative NtcA binding site (located at -370 bp to -151 bp, relative to the tsp). The mobility shift assays were performed using: two unspecific DNA fragments (II and IV), obtained by PCR amplification of the multiple cloning sites of the plasmids pQE-30 (Qiagen) and pBluescript SK+ (Stratagene), respectively; part of the promoter region of *hupSL *(III), and different amounts of purified NtcA. The NtcA-*hupSL *promoter complexes are indicated as I.

**Figure 3:**
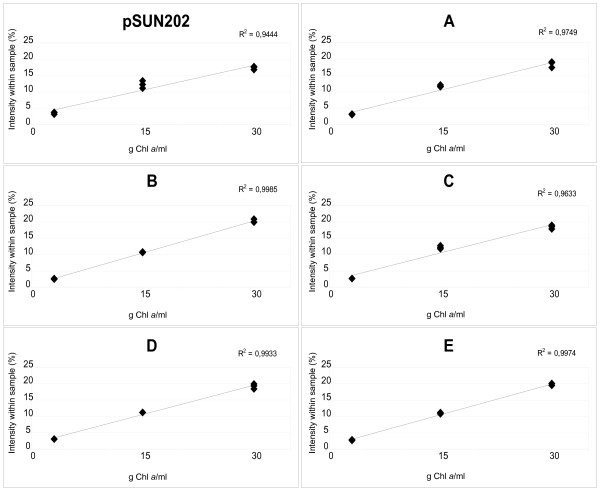
**Optimization of GFP fluorescence measurements**. The curves demonstrate GFP intensity within a culture of transformed *Nostoc punctiforme *ATCC 29133. The cultures have been transformed with a self replicative vector, pSUN202, where truncated versions of the *hupSL *promoter have been fused to *gfp *(constructs A to E). Dilutions of the cultures, ranging from 3–30 μg Chl *a*/ml, have been plotted against the intensity (%). All dilutions have been measured in triplicates and the total fluorescence in the sample is 100%.

To elucidate if NtcA binds to the identified NtcA binding site (TGT-N_9_-ACA), centred at 258.5 bp upstream the tsp (Fig. 1), in the *hupSL *promoter, Electrophoretic Mobility Shift Assays (EMSA), using the *hupSL *promoter from *N.punctiforme *and NtcA protein from *Nostoc *PCC 7120, were performed (Fig. 2). The result showed that NtcA does indeed interact with the *hupSL *promoter and retard it on the gel. Two unrelated DNA fragments (335 bp and 229 bp, respectively), with no known NtcA binding sites showed no interaction with NtcA (Fig. 3). This demonstrates the specificity of the binding of NtcA to the 241 bp *hupSL *promoter fragment.

### Generation of *hupSL *reporter gene constructs

To define and identify regulatory regions in the promoter controlling *hupSL *transcription a deletion analysis of the promoter was carried out. Five *hupSL *promoter sequences of various lengths (A-E; Fig. 1) were cloned by PCR and coupled to *gfp*, encoding the reporter protein GFP, or to *luxAB *encoding the reporter enzyme Luciferase (Fig. 1). The lengths of the truncated promoter fragments were designed according to the positions of the putative binding sites for Integration Host Factor (IHF) and NtcA, identified in the *hupSL *promoter using bioinformatics (Fig. 1) [14].

### Confirmation of the insertion of correct promoter deletions constructs

Cells from *N. punctiforme *were transformed by electroporation with vector constructs containing various lengths of the *hupSL *promoter coupled to *gfp *(A-E) or *luxAB *(1–5) (Fig. 1). Positive clones were confirmed by colony PCR. The primers used for the colony PCR anneal to the vector sequences flanking the inserted promoter region and hence the product spans the full length of the insert (Table 1). Analysis of the obtained results indicates that all the cloned fragments were of a length expected for the correct construct (data not shown).

### Optimization of GFP fluorescence measurements

To be able to compare the GFP expression from the different promoter deletions, dilution series were made to confirm that measurements were done in a range where the GFP signal are linear for all the constructs. The curves show high R^2 ^values, ranging between 0.96 to 1.0, confirming that there is only very little or no saturation of the signal using the cell density chosen for the measurements (assessed by Chlorophyll *a *concentration) (Fig. 3). Experiments with dilution series of the bioluminescence measurements showed high R^2 ^values ranging from 0.79 – 0.99

### Expression from the *hupSL *promoter deletions

**Figure 4:**
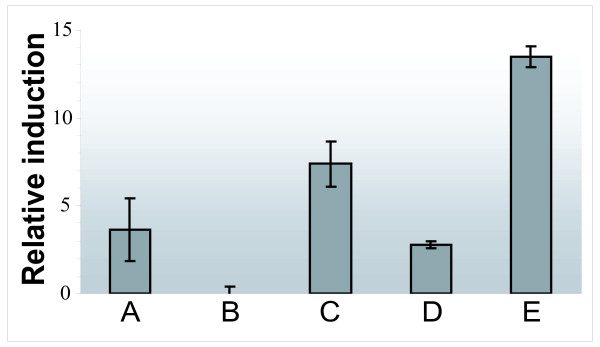
**Expression from the *hupSL *promoter deletions**. Measurements of GFP fluorescence intensity in living cells grown under nitrogen fixing conditions. *Nostoc punctiforme *ATCC 29133 cells were transformed with vector constructs containing truncated versions of the *hupSL*-promoter (A-E) fused to the reporter gene *gfp *(see Figs. 1 and 2). All values are normalised to the expression from the promoter less reporter vector, pSUN202 (negative control) and the GFP intensity is shown as relative intensity compared to the negative control. All measurements were performed in triplicates.

The measurements of GFP intensity and hence promoter activity were performed on living cells grown under nitrogen fixing conditions. The shortest promoter fragment, E, (stretching from -57 to tsp), showed the highest expression level (Fig. 4) in all experiments. This was also confirmed in the measurements of bioluminescence, where construct E showed the highest expression levels (data not shown). This part of the *hupSL *promoter lacks the putative IHF and NtcA binding sites (Fig. 1). There were minor variations between the promoter activities of the four longer promoter fragments (construct A-D). The loss of the most upstream putative IHF seems to have a slight positive effect on transcription (construct C) and the loss of the NtcA binding site seems to have a slight negative effect on transcription (construct D). Furthermore, in some of the experiments the promoter activity was almost abolished for construct B, while other experiments showed only a low activity. The part of the promoter retained in construct A but lost in construct B contains no known putative binding sites for transcriptional regulators. It should be noted that the differences of expression between the longer promoter fragments (constructs A-D) were significant within experiments (three independent measurements) but not always between the experiments. However, all experiments showed the same general expression pattern for fragments A-D even though the actual levels differed. The difference between the longer promoter fragments (construct A-D) and the shortest fragment (construct E) were significant between all experiments. As expected, the positive control p*PrbcL-gfp *showed very high expression levels in all experiments (data not shown).

### *In situ *localization of *hupSL *transcript

**Figure 5:**
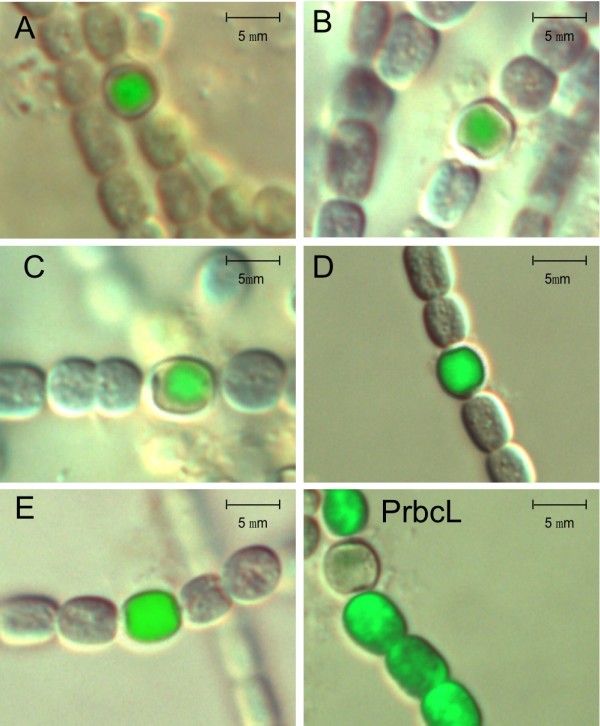
***In situ *localization of *hupSL *transcript**. Micrographs showing localization of the GFP expression from the *hupSL *promoter in nitrogen fixing filaments of *Nostoc punctiforme *ATCC 29133. *N. punctiforme *cells were transformed with a self replicative vector, pSUN202, containing deletions of the *hupSL *promoter fused to *gfp *(see Fig. 1). The micrographs were constructed by merging the DIC image with the corresponding fluorescence image for all promoter constructs (A to E, see Fig. 1) and the control construct p*PrbcL-gfp*. The green color in the micrographs has been enhanced digitally to make pictures clearer and the degree of enhancement differ for different constructs.

To investigate if the truncated parts of the *hupSL *promoter, except from being important for the expression levels, also affected the cellular localization of *hupSL *transcription fluorescence microscopy was used to view the living cells. Furthermore, this study was carried out to analyze if the high transcription level of the shortest promoter fragment (construct E, promoter fragment stretching from -57 to tsp) was the result of a general low expression in all cells rather than high specific expression in the heterocyst. Images of the filaments were taken using bright field and fluorescence microscopy and then merging the images. The micrographs showed that promoter fragments A-D had heterocyst specific expression (Fig. 5). Surprisingly, even the shortest promoter construct E showed a heterocyst specific expression (Fig. 5). The promoter region of *PrbcL *fused to *gfp*, used as a positive control, gave, as expected, high expression primarily in vegetative cells [49,50] (Fig. 5).

## Discussion

The transcriptional regulation of *hupSL*, encoding the cyanobacterial uptake hydrogenase, has here been examined in the heterocystous, nitrogen fixing cyanobacterium *Nostoc punctiforme *ATCC 29133. The promoter has been characterized by fusing truncated versions of the *hupSL *promoter to reporter genes. In this study we have chosen to use two different types of reporter genes, *gfp *and *luxAB*, encoding GFP and luciferase respectively. GFP, unlike luciferase, has the advantage that it does not require addition of a substrate, which eliminates toxicity and permeability problems [47]. On the other hand GFP, unlike luciferase, is a very stabile protein and tend to accumulate in the cells [51]. In addition, it has been reported that different reporter genes may give very different patterns of expression for a single promoter if these promoters are sensitive to DNA topology [52]. Similarly, it was shown that the CAT reporter system exerts unusual effects on various gene promoters, including silencer activities, which did not represent the true regulatory mechanisms [53]. To strengthen the results of the study, and to avoid drawing conclusions about anomalies occurring from studying the expression of an exogenous protein, both reporter systems were used in parallel in this study.

Putative binding sites of NtcA have been identified and confirmed in the *hupSL *promoter of several cyanobacteria. The NtcA binding site identified in *N. punctiforme *differs from the optimal consensus NtcA binding site (GTAN_8_TAC) usually found in NtcA activated promoters. These NtcA activated promoters contain an *E. coli *like σ^70 ^-10 box and the NtcA site is centred approximately 41.5 bp upstream the tsp where an *E. coli *σ^70 ^like -35 box is usually found [16]. These characteristics makes the NtcA activated promoters similar to class II, CAP-activated, promoters [16]. However the NtcA consensus sequence identified in *N. punctiforme *(TGT-N_9_-ACA) has also been reported for several other promoters, for example in promoters of *rbcL*, *xisA *and *gor *in *Nostoc *sp. PCC 7120 [54] and for *hupSL *in *A*. *variabilis *ATCC 29413 [35] and is believed to represent a weaker binding site [54]. The binding of NtcA to the TGT-N_9_-ACA consensus binding sequence in the *hupSL *promoter has been shown in *A.variabilis *[35] and was also demonstrated here for *N. punctiforme *(Fig. 2). NtcA bound specifically to a 241 bp DNA fragment of the *N. punctiforme hupSL *promoter containing the putative NtcA binding site. In a recent study, using an *ntcA *mutant, the *hupSL *expression in *A. variabilis *was shown to be directly, or indirectly, regulated by NtcA [35]. However, the importance of the NtcA binding site for *hupSL *transcription in *N. punctiforme *seems to be rather low (Fig. 5) since the presence or absence of the NtcA binding site in the *hupSL *promoter had no major effect on the transcription of GFP, or Luciferase, in the promoter deletion study presented here. In the *hupSL *promoter of *N. punctiforme *the putative NtcA binding site is located quite far upstream of the tsp (centered at -258.5 bp) (Fig. 1). NtcA binding sites at distances greater than given for NtcA activated promoters have been reported earlier [15]. However, it can not be excluded that this NtcA binding site is not regulating *hupSL *transcription, but instead the transcription of the gene of unknown function located upstream of *hupS*, Npun_R0367. This gene is located in the opposite DNA strand compared to *hupSL *and the putative NtcA binding site is centred at 234.5 bp upstream the translation start site of Npun_R0367 (Fig. 1). A recent study has suggested this ORF to encode a protein involved in the maturation of the small subunit, HupS, of cyanobacteria hydrogenases [10]. A regulation of this gene by NtcA would therefore not be unlikely. The regulation of *hupSL *expression differs between different strains of cyanobacteria. For example in *A. variabilis *ATCC 29413, a strain expressing an alternative vanadium containing nitrogenase during molybdenum limiting conditions [55], and a second Mo-depending nitrogenase both in heterocysts and vegetative cells during anaerobic conditions [56], a low expression of *hupSL *could be detected in vegetative cells. Furthermore, *hupSL *transcription has been shown to be upregulated by the presence of H_2 _in some *Nostoc *strains [33,34] but not in *A. variabilis *[35]. Due to these differences between strains variations in the regulation of *hupSL *transcription between *A. variabilis *ATCC 29413 and *N. punctiforme *are expected.

The differences in promoter activity between promoter fragments A-D, (Fig. 4) were not always significant between the experiments. However, when comparing different experiments, the same overall expression patterns were seen. One explanation for the variation of expression between experiments for construct A-D could be e.g. slight variations in age of the heterocysts between the experiments. Due to this variation between experiments one should be careful in making conclusions about the importance of these differences in transcription levels between constructs A – D. Looking at individual experiments, presence of the NtcA binding site combined with the loss of the most upstream putative IHF binding, site seem to have a slight positive effect on transcription, as well as the loss of the most downstream IFH binding site. There is also room to speculate that there is some positive regulation located upstream the previously identified putative binding sites in the *hupSL *promoter (Figs. 1 and 5), however further experimental studies, with for example directed mutagenesis, are necessary. This is also true for promoter fragment B, which unique part contains no known putative binding sites for transcriptional regulators. Still, in some experiments the promoter activity was abolished while others showed only a low activity – a finding that deserves further attention.

In this paper we have shown that the part of the *hupSL *promoter region that gave the highest expression level is limited to a 316 bp DNA fragment stretching from -57 (in relation to tsp) to the translation start site (Fig. 4). Not only does this short promoter confer a high transcription level, it also retains heterocyst specificity. A loss of heterocyst specificity could have lead to a misleading conclusion of high promoter activity: the promoter would have shown high total expression, due to expression in all cells, even if the promoter activity was still low. However the fact that this promoter fragment kept heterocyst specificity (Fig. 5) enables us to draw the conclusion that the activity of the shortest promoter is truly higher than for the other promoter fragments. One assumption could be that heterocyst specificity of expression is due to a transcription factor binding to the *hupSL *promoter and stimulating transcription in heterocysts. However, another possibility could be that *hupSL *is constitutively transcribed and that vegetative cells contain a repressor lacking in heterocysts which restrain transcription in that cell type. If the heterocyst specificity is mediated by an activator binding the short promoter sequence upstream the tsp (or perhaps the untranslated leader region downstream the tsp) or by a repressor only present in vegetative cells needs to be subjected to further investigations. Further characterization of this short promoter region will not only give information about what promotes *hupSL *transcription but can also help answering the question what directs heterocyst specific expression of genes and pattern formation in *N. punctiforme*, and perhaps other heterocystous, filamentous cyanobacteria.

## Conclusion

The result that the 57 bp promoter is a highly active promoter is most interesting and will be investigated further. This short DNA sequence, and its 258 bp untranslated leader region downstream the tsp, appears to harbour enough information to make the transcription to occur in heterocysts only. Taken one step further, if this information conferring heterocyst specific transcription can be elucidated it will give clues to what signals are involved in heterocyst specific gene expression and pattern formation in filamentous cyanobacteria.

## Authors' contributions

MH performed most experimental work; promoter constructs transformation of *Nostoc *cells, fluorescence and luminescence measurements and documentations. She was the primary author of the final manuscript. PO and PiL carried out the EMSA experiments and were involved in the planning of the experiments, analyses of the data, and writing the manuscript. KS supervised the experimental work and analyses of the data, and was also involved in all parts of the writing of the manuscript. PeL conceived and coordinated the project, and the manuscript. All authors have read and approved the manuscript.
